# Salicylic acid remodeling of the rhizosphere microbiome induces watermelon root resistance against *Fusarium oxysporum* f. sp. *niveum* infection

**DOI:** 10.3389/fmicb.2022.1015038

**Published:** 2022-09-23

**Authors:** Feiying Zhu, Yong Fang, Zhiwei Wang, Pei Wang, Kankan Yang, Langtao Xiao, Ruozhong Wang

**Affiliations:** ^1^Hunan Academy of Agricultural Sciences, Changsha, China; ^2^Hunan Provincial Key Laboratory of Phytohormones, College of Bioscience and Biotechnology, Hunan Agricultural University, Changsha, China

**Keywords:** salicylic acid, rhizosphere, microbiome, watermelon, *Fusarium* wilt

## Abstract

*Fusarium* wilt disease poses a severe threat to watermelon cultivation by affecting the yield and quality of the fruit. We had previously found that the rhizosphere microbiome has a significant impact on the ability of watermelon plants to resist *Fusarium* wilt development and that salicylic acid (SA) is closely related to this phenomenon. Therefore, in this study, the role of SA as a mediator between plants and microbes in activating resistance against *Fusarium oxysporum* f. sp. *niveum* (FON) infection was explored through physiological, biochemical, and metagenomic sequencing experiments. We demonstrated that exogenous SA treatment could specifically increase some beneficial rhizosphere species that can confer resistance against FON inoculation, such as *Rhodanobacter*, *Sphingomonas*, and *Micromonospora*. Functional annotation analysis indicated that SA application significantly increased the relative abundance of glycoside hydrolase and polysaccharide lyase genes in the microbiome, which may play an essential role in increasing plant lipids. Moreover, network interaction analysis suggested that the highly expressed *AAC6_IIC* gene may be manipulated through SA signal transduction pathways. In conclusion, these results provide a novel strategy for controlling *Fusarium* wilt in watermelons from the perspective of environmental ecology, that is, by manipulating the rhizosphere microbiome through SA to control Fusarium wilt.

## Introduction

Watermelon (*Citrullus lanatus*) is an important horticultural crop worldwide (Everts and Himmelstein, 2015). However, the commercial cultivation of this fruit is severely threatened by the soil-borne fungus *Fusarium oxysporum* f. sp. *niveum* (FON), which causes *Fusarium* wilt, a disease that leads to a significant decline in crop quality and yield (Xu et al., 2015; Ren et al., 2016; Li et al., 2019). Salicylic acid (SA), a phytohormone present in plants and some microbes, is reported to be an important signaling molecule that induces plant resistance to diseases (Yang et al., 2015; Zhang and Li, 2019). With the deciphering of the watermelon genome, Lü et al. found *via* gene ChIP transcription analysis that the phenylalanine ammonia lyase (*PAL*) gene plays an important role in the lignin metabolic pathway of resistance to *Fusarium* wilt (Lü et al., 2011). Lv reported that the intercropping of wheat and watermelon with FON induced SA synthesis in the watermelon plant to enhance its resistance to *Fusarium* wilt (Lv et al., 2018). Furthermore, our research demonstrated the important role of SA in regulating watermelon resistance against *Fusarium* wilt, with results indicating that the significantly expressed *C. lanatus PAL* (*ClPAL*) and non-pathogen-related (*NPR*) genes play key roles in SA synthesis and signal transduction in this plant species (Zhu et al., 2022a,b).

Recently, increasing evidence has suggested that the rhizosphere microbiome plays critical roles in promoting plant growth and health, such as enhancing nutrient uptake by the host plant and increasing its resistance against pathogen attack (Levy et al., 2017; Berendsen et al., 2018). The establishment of plant–rhizosphere microbiome interaction is a highly coordinated event influenced by the host plant and soil. For instance, the plant immune system shapes the microbiome, which, in turn, can increase the plant’s immune capacity (Heintz and Mair, 2014; Levy et al., 2017; Aleklett et al., 2018). Notably, our previous studies have demonstrated the important role of the soil microbial community structure in controlling the occurrence of *Fusarium* wilt in watermelons (Zhu et al., 2018, 2019). For instance, our results indicated that the presence of beneficial microbes, such as *Rhodanobacter*, *Pseudomonas*, *Sphingomonas*, and *Herbaspirillum*, is important for the prevention of watermelon disease (Zhu et al., 2020).

Consistently, more researchers have begun to notice that rhizosphere microbiome composition is influenced by an array of plant-derived metabolic substances (Trivedi et al., 2020; Liu et al., 2021). Additionally, the essential role of SA in recruiting specific rhizosphere microbiomes has been previously reported. For instance, Trivedi et al. showed that SA affected the abundance of specific bacterial groups in the roots *via* a combination of direct and indirect effects (Trivedi et al., 2020). However, the mechanism by which watermelon plants affect microbiome assembly and the impact of this interconnectedness on plant and microbiota functions remains unclear. Chen et al. found that systemic accumulation of SA could affect microbiome assembly in the rhizosphere of *Arabidopsis* plants after foliar infection by pathogens (Chen et al., 2020). Therefore, through metagenomic sequencing and relevant physiological and biochemical analyses, we aimed to explore the effect of exogenous SA in remodeling the watermelon rhizosphere microbiome to induce resistance against FON infection. Our research findings provide a new strategy for controlling watermelon *Fusarium* wilt from the perspective of environmental ecology and have significant value for promoting sustainable agricultural development.

## Materials and methods

### Experimental process and sampling

The experiment was conducted in the city of Changsha (112°58′42″E, 28°11′49″N), Hunan Province, China. The soil used for planting was sandy loam, collected from our field experiment at the Gaoqiao Scientific Research Base of the Hunan Academy of Agricultural Sciences in Changsha (Zhu et al., 2020). Our previous studies found that the high content of pathogens in the soil under a continuous cropping system leads to SA accumulation in the plant (Zhu et al., 2022a,b). Therefore, we sterilized the soil to make them as same background before use (LDZM-80KCS-3 vertical pressure steam sterilizer, ZHONGAN, Shanghai, China) to avoid errors and study how SA can improve watermelon immune resistance at the early stage after FON infection. The watermelon variety used was Zaojia 8,424 (Xinjiang Farmer Seed Technology Co., Ltd., Urumqi, China), which is the main cultivar on the Chinese market. The watermelon seedlings were cultivated in seedling pots containing peat, perlite, and vermiculite (6:3:1) and grown in a biochemical incubator (LRH-300, ZHUJIANG, Taihong, Shaoguan, China) that was set at 25°C during 16 h of light and 18°C during 8 h of darkness. After 30 days, each plant was transplanted into separate pots. When the seedlings were at the two-leaf stage, 5 ml of 100 μM exogenous SA (Sigma-Aldrich LLC., Merck KGaA, Darmstadt, Germany) was incorporated into the root zone of each plant, and another 5 ml was added 24 h later. The Fusarium strain FON was firstly incubated in the dark for 7 days on a PDA plate at 28°C. Then, a bam plug was selected from the PDA plate and placed into 300 ml of potato dextrose broth in a flask, before propagation on a rotary shaker at 200 rpm at 26–30°C. Two days later, 5 ml FON (1 × 10^6^ conidia/mL) was added to the root zone of each plant (Li et al., 2019).

The plants were divided into the following three groups: S, mock-inoculation control (H_2_O); SA, 100 μM exogenous SA + FON; and SF, FON only. We set six different sampling times before and after the different treatments: 0 days post-inoculation (dpi) (before treatment), 12 h post-inoculation (hpi), 1 dpi, 3 dpi, 5 dpi, and 7 dpi. Sampling was stopped at 7 dpi when the disease symptoms (yellowing and wilting) started to appear. We selected 10 watermelon plants as one replicate and set three independent replicates (30 plants) for each sample group, at six different sampling times with every three treatments (16 groups). Therefore, 480 plant samples were collected from 480 pots.

The soil samples were designated S0 (mock-inoculation control, before treatment), S3 (mock-inoculation control, 3 dpi), SF3 (FON treatment, 3 dpi), SA3 (SA + FON treatment, 3 dpi), S7 (mock-inoculation control, 7 dpi), SF7 (FON treatment, 7 dpi), and SA7 (SA + FON treatment, 7 dpi). For each treatment group, the rhizosphere soil sample was pooled from 10 plant roots in one repetition, with three independent replicates, at each sampling time. After removing the plant roots and stones, the rhizosphere soil samples were placed in 5 ml sterile centrifuge tubes and then divided into three parts. In total, 21 samples were collected at the three different sampling times (i.e., 0 dpi, 3 dpi, and 7 dpi) and stored at −80°C for sequencing analysis.

### Determination of the watermelon plant root morphology and physiological and biochemical indexes

First, the root morphology of each plant was captured using a digital camera. The roots were then cut with sterilized scissors, and their fresh weights were measured using an electronic balance. Thereafter, the roots were placed in sterilized 5 ml centrifuge tubes to test their peroxidase (POD) and phenylalanine ammonia lyase (PAL) activities and malondialdehyde (MDA) content. The POD and PAL activities were analyzed using a BC0095 peroxidase assay kit and BC0215 PAL test kit (Beijing Solarbio Science & Technology Co., Ltd., Beijing, China), respectively, whereas the MDA content was determined using the thiobarbituric acid method using a BC0025 MDA assay kit (Beijing Solar-bio Science & Technology Co., Ltd.), according to the manufacturer’s protocols. A Tecan-SPARK microplate reader (Tecan Trading AG, Männedorf, Switzerland) and Eppendorf 5415R refrigerated centrifuge (Eppendorf AG, Hamburg, Germany) were used for the assays. Three biological replicates per sample were performed, with three technical replicates.

The disease incidence was calculated as follows:


$$\begin{array}{l} \mathrm{Dise}a\mathrm{se}\;\mathrm{incidence}\left(\%\right)=(\mathrm{no}.\mathrm{of}\;\mathrm{infected}\;\mathrm{plants}\\ \mathrm{/ total}\;\mathrm{number}\;\mathrm{of}\;\mathrm{plants}\;\mathrm{surveyed)}\times 100. \end{array}$$

### Soil DNA library preparation

A total of 1 μg DNA per sample was used for sequencing. In brief, DNA sequences 350 bp in size were fragmented by sonication, and the fragments were then end-polished, A-tailed, and ligated with the full-length adaptor for Illumina sequencing followed by PCR amplification. The PCR products were purified (AMPure XP system), and libraries were prepared and analyzed for size distribution using the Agilent2100 Bioanalyzer. Three biological replicates per sample were analyzed. Raw data were obtained using the Illumina NovaSeq 6000 sequencing platform.

### Metagenomic sequencing

Readfq V8 was used to acquire the clean data for subsequent analysis against the host database using the Basic Local Alignment Search Tool (BLAST), which uses Bowtie 2.2.4 as default software to filter the reads that are of host origin. After all the reads that were not used in the forward step were combined, SOAPdenovo was used to generate the mixed assembly using the same parameters as those applied for the single assembly. The Scaftigs (≥500 bp) assembled from both the single and mixed assemblies were used to predict the open reading frames (ORFs) using MetaGeneMark software, and sequences shorter than 100 nt were filtered from the predicted result with default parameters. For ORF prediction, sequence redundancy was reduced using the Cluster Database at High Identity with Tolerance software, and a unique initial gene catalog was obtained. The clean data of each sample were mapped to the initial gene catalog using Bowtie 2.2.4 with the following parameter settings: --end-to-end, −-sensitive, -I 200, and -X 400. Genes with two or fewer reads in each sample were filtered, and the gene catalog (UniGene database) obtained was used for subsequent analysis.

The abundance of each gene in each sample was statistically analyzed based on the number of mapped reads and their lengths. The basic information statistics, core- and pan-genomic analyses, correlation analysis of the samples, and Venn diagram analysis of the number of genes were all based on the abundance of each gene in each sample in the gene catalog. DIAMOND software was used to blast the UniGene database to the sequences of bacteria, fungi, archaea, and viruses, which were all extracted from the non-redundant database of the National Center for Biotechnology Information (NCBI). Finally, the clean reads were deposited in the NCBI Sequence Read Archive database (Accession number: PRJNA707127).

### Assembly of the Core rhizosphere communities, common functional databases used, and resistance gene annotation

To determine the dynamic changes in the dominant soil microbial communities during all three sampling times in the different treatment groups, we used community bar-plot analysis to identify the 10 most abundant soil microbial communities at both the phylum and genus levels. Moreover, we used multiple *t*-tests to compare significant differences in soil microbial communities at 3 and 7 dpi, both at the phylum and genus levels, after SA application. To elucidate the molecular mechanism by which the rhizosphere microbial community cooperates to induce plant resistance against *Fusarium* wilt, we blasted the unique genes against various functional annotation databases such as the Kyoto Encyclopedia of Genes and Genomes (KEGG), Evolutionary Genealogy of Genes: Non-supervised Orthologous Groups (eggNOG), Carbohydrate-Active Enzymes (CAZy), and the Comprehensive Antibiotic Resistance Database (CARD) to determine their abundance and analyzed them statistically with a visual display.

KEGG, eggNOG, and CAZy databases were used for functional annotation of the resistance genes. The Unigenes were aligned to the CARD database using Resistance Gene Identifier software with default parameter settings. The relative abundance of the antibiotic resistance ontology (ARO) cluster, abundance bar charts, abundance cluster heatmaps, and differences in the number of resistance genes between soil groups were displayed according to the alignment results. Similarly, the distribution of resistance genes in each sample and analyses of the species attribution of those genes and their resistance mechanisms were also investigated.

### Statistical analysis

Statistical analysis was performed using GraphPad Prism 9 (GraphPad Software, San Diego, CA, United States). All values are expressed as mean ± standard error (*n* = 3). The differences between the groups were tested using an analysis of similarities. Figures were constructed using Microsoft Office 2010 (Microsoft Corporation, Redmond, WA, United States).

## Results

### Effectiveness of salicylic acid treatment in controlling *fusarium* wilt of watermelon

**Figure 1:**
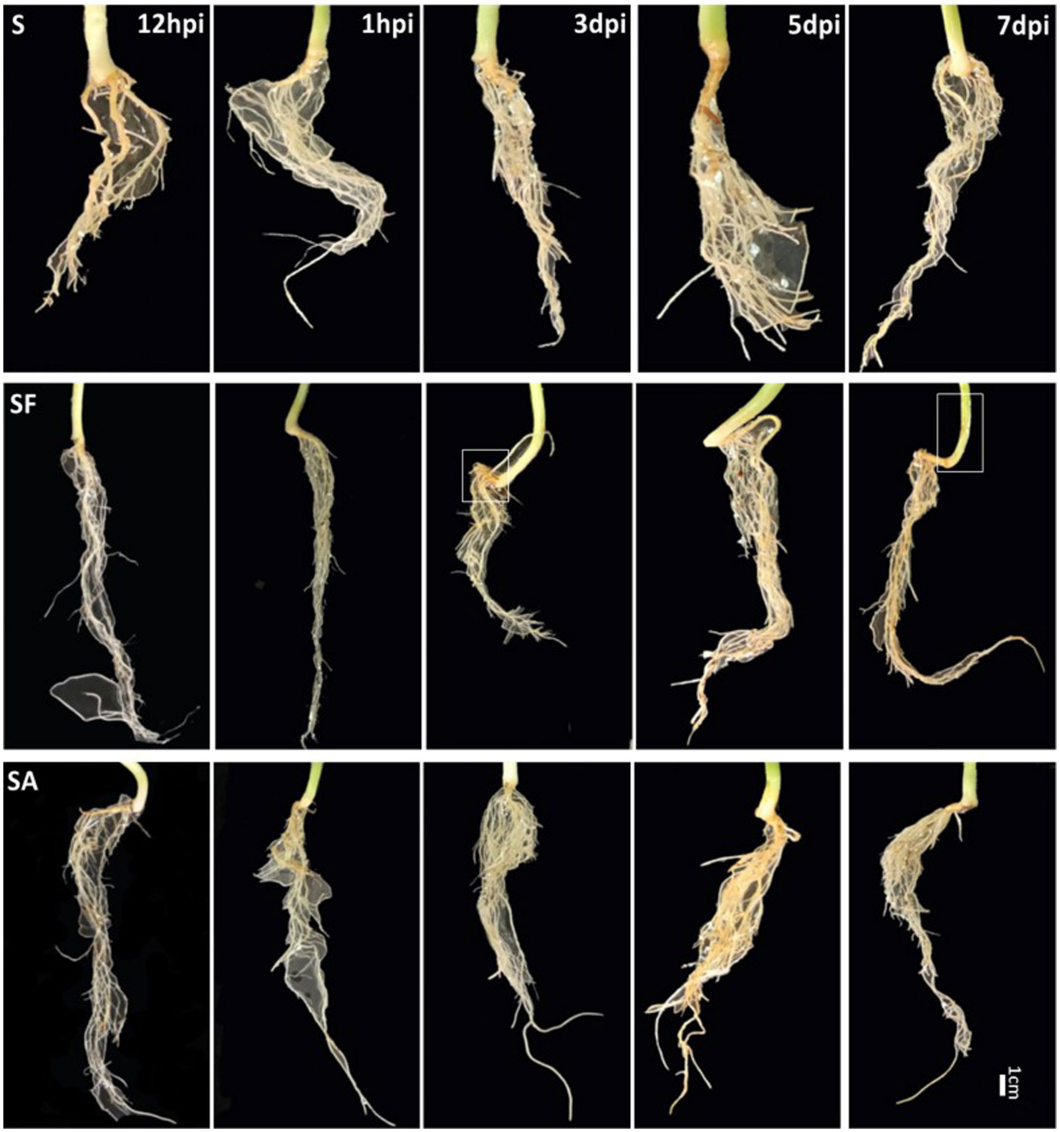
Comparison of watermelon root phenotypes in different treatment groups S, Mock-inoculation control; SA, SA + FON treatment; SF, FON treatment; FON, *Fusarium oxysporum* f. sp. *niveum*; SA, salicylic acid; hpi, hours post-inoculation; dpi, days post-inoculation.

**Figure 2:**
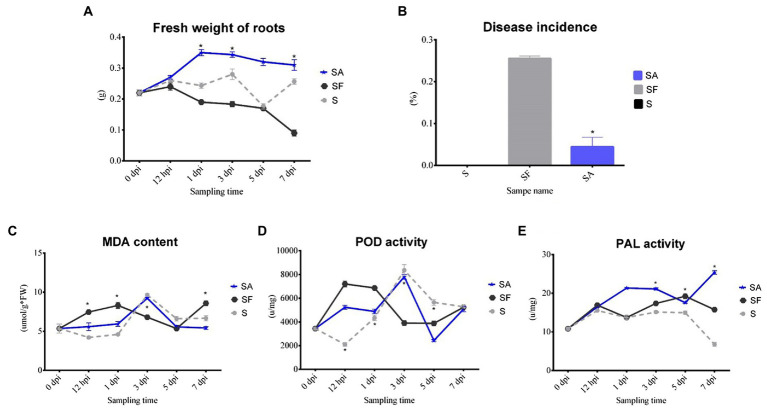
Comparison of physiological changes, disease incidence, and biochemical indexes of watermelon plants after exogenous salicylic acid application. **(A)** Comparison of the root fresh weight of different samples. **(B)** Comparison of the disease incidence in different samples. **(C)** Comparison of the malondialdehyde (MDA) content in different samples. **(D)** Comparison of the peroxidase (POD) activity in different samples. **(E)** Comparison of the phenylalanine ammonia lyase (PAL) activity in different samples. S, Mock-inoculation control; SA, SA + FON treatment; SF, FON treatment; FON, *Fusarium oxysporum* f. sp. *niveum*; SA, salicylic acid; hpi, hours post-inoculation; dpi, days post-inoculation; 0 dpi: before treatment. Three biological replicates per sample were analyzed. Data are expressed as mean ± SE (*n* = 3). Multiple *t*-tests of ANOSIM (*p ≤ 0.0001).

To clarify whether exogenous SA treatment can control *Fusarium* wilt disease in watermelon, we first compared the root phenotypes after exposure to exogenous SA + FON or FON alone at five different sampling times. The SA group had more fibrous roots than the SF group did. The roots in the SF group began to turn yellow at 3 dpi, and obvious plant yellowing and wilting symptoms were observed at 7 dpi (Figure 1). Furthermore, there was a significant difference in the fresh weight of the roots between the samples at 1, 3, and 7 dpi. For instance, the root fresh weight of the SA group was 2.5 times heavier than that of the SF group at 7 dpi (Figure 2A). The incidence of *Fusarium* wilt after SA application was significantly lower (by –20%) than that in plants not treated with phytohormones (Figure 2B). Moreover, the MDA content in the SF group first increased from 12 hpi onward, then declined at 3 dpi and finally increased significantly again at 7 dpi. However, we noticed an increase in MDA content at 3 dpi in both SA and S groups (Figure 2C). Similarly, POD activity was significantly increased at 3 dpi in the SA and S groups, which indicated that this sampling time might be a key time point for activating resistance defenses in watermelon plants (Figure 2D). Moreover, there were significant enhancements in PAL activity in the SA group at 3 dpi and 7 dpi, whereas the enzyme activity was significantly lower in the SF group at 7 dpi (Figure 2E), which confirmed the symptoms and disease incidence results. Therefore, the results from plant root physiological and biochemical studies indicate that exogenous SA treatment can effectively reduce the incidence of *Fusarium* wilt in watermelon.

### Sequencing and metagenome assembly

We selected three sampling times to compare the dynamic changes in the dominant soil microbial communities between the different treatments. Gene sequencing analysis revealed that the ORFs were approximately 200–600 nt in length (Supplementary Figure S1A). According to the results of the quality control analysis, the evenness of the number of ORFs observed in the samples tended to be consistent (Supplementary Figure S1B). The Venn diagram showed that there were 624,730 overlapping genes among all groups of samples, and there were unique genes in each group (Supplementary Figure S1C).

### Dynamic changes in the soil microbial community structure

**Figure 3:**
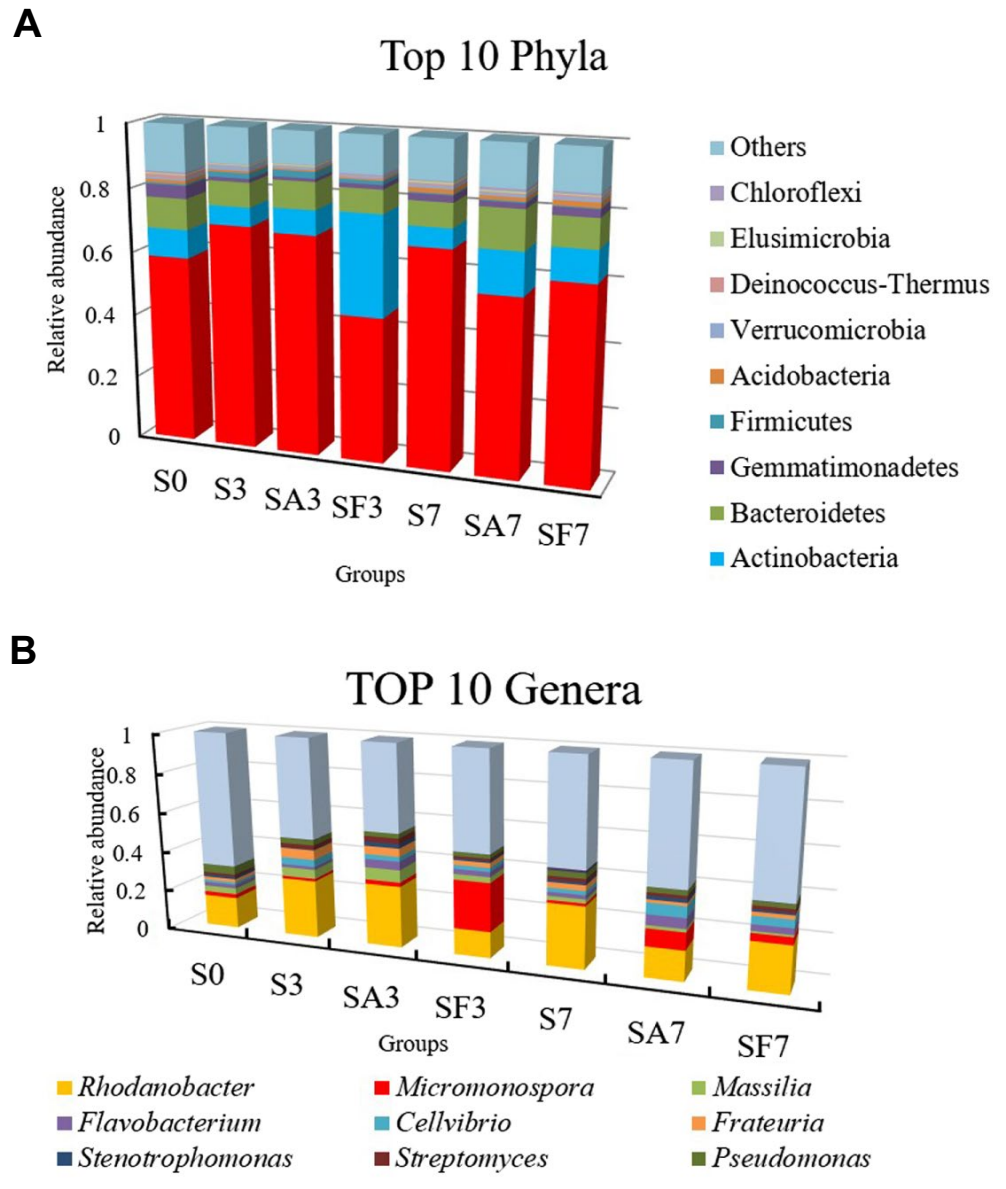
Assembly of core rhizosphere microbial communities. **(A)** Community bar-plot analysis of the relative abundance of rhizosphere microbial communities in the different soil groups at the phylum level. **(B)** Community bar-plot analysis of the relative abundance of rhizosphere microbial communities in the different soil groups at the genus level. S0, Mock-inoculation control (before treatment); S3, mock-inoculation control, 3 dpi; S7, mock-inoculation control, 7 dpi; SA3, SA + FON treatment, 3 dpi; SA7, SA + FON treatment, 7 dpi; SF3, FON treatment, 3 dpi; SF7, FON treatment, 7 dpi. FON, *Fusarium oxysporum* f. sp. *niveum*; SA, salicylic acid; dpi, days post inoculation. Three biological replicates per sample were analyzed. Data are expressed as mean ± SE (*n* = 3).

**Figure 4:**
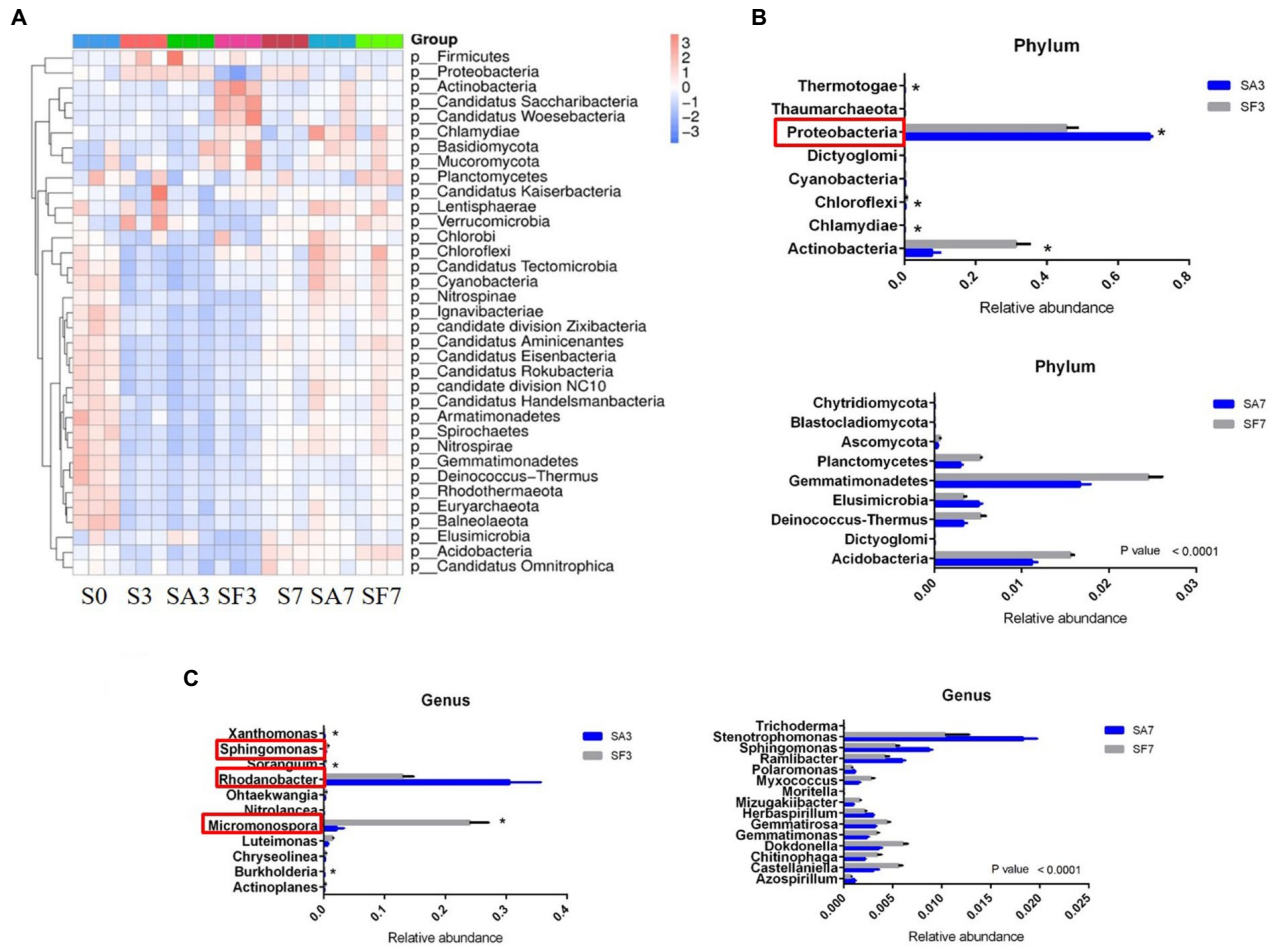
Dynamic changes in the significantly abundant rhizosphere microbial communities. **(A)** Heatmap of the dynamic changes in different phyla among the various soil groups. **(B)** Bar plots showing the significant differences in relative abundance of the main phyla. **(C)** Bar plots showing the significant differences in relative abundance of the main genera. Three biological replicates per samples were analyzed. Data are expressed as mean ± SE (*n* = 3). Multiple *t*-tests (*p ≤ 0.01). S0, Mock-inoculation control, before treatment; S3, mock-inoculation control, 3 dpi; S7, mock-inoculation control, 7 dpi; SA3, SA + FON treatment, 3 dpi; SA7, SA + FON treatment, 7 dpi; SF3, FON treatment, 3 dpi; SF7, FON treatment, 7 dpi; FON, *Fusarium oxysporum* f. sp. *niveum*; SA, salicylic acid; dpi, days post-inoculation.

The top 10 phyla in all samples were Proteobacteria, Actinobacteria, Bacteroidetes, Gemmatimonadetes, Firmicutes, Acidobacteria, Verrucomicrobia, Deinococcus-Thermus, Elusimicrobiota, and Chloroflexi (Figure 3A). The top 10 genera were *Rhodanobacter*, *Micromonospora*, *Massilia*, *Flavobacterium*, *Cellvibrio*, *Frateuria*, *Stenotrophomonas*, *Streptomyces*, *Pseudomonas*, and *Solimonas* (Figure 3B). Additionally, the heatmap showed dynamic changes in the rhizosphere communities among the groups at different sampling times; that is, there was an obvious enrichment of Proteobacteria and Firmicutes in the SA3 sample but a higher accumulation of Actinobacteria, Candidatus Saccharibacteria, Candidatus Woesebacteria, Chlamydiae, and Mucoromycota in the SF3 sample (Figure 4A). In agreement with previous research, our results indicated that Proteobacteria is the second largest phylum of hydrogenogenic CO oxidizers, which may play a significant role in helping plants against FON infection (Badger and Bek, 2008; Wang and Sugiyama, 2020). Overall, our results indicated that the application of SA changed the watermelon rhizosphere soil microbial communities.

For instance, at the phylum level, the abundances of Actinobacteria, Chlamydiae, Chloroflexi, Cyanobacteria, Dictyoglomi, Proteobacteria, Thermotogae, and Thaumarchaeota differed significantly between the SA3 and SF3 samples (Figure 4B). Moreover, the SA3 sample showed a significant abundance of Proteobacteria, whereas the SF3 sample was significantly enriched with Actinobacteria. In contrast, the SA7 sample had a significantly higher abundance of Blastocladiomycota, Dictyoglomi, and Elusimicrobiota, whereas the SF7 sample had a high abundance of Actinobacteria, Dictyoglomi, Gemmatimonadetes, Deinococcus-Thermus, Ascomycota, and Planctomycetes (Figure 4B). At the genus level, the abundance of *Rhodanobacter* increased significantly in the SA3 sample, whereas that of *Actinoplanes*, *Burkholderia*, *Chryseolinea*, *Luteimonas*, *Micromonospora*, *Nitrolancea*, *Ohtaekwangia*, *Sorangium*, *Sphingomonas*, and *Xanthomonas* decreased significantly compared to the levels in the SF3 sample (Figure 4C). However, the abundances of *Stenotrophomonas, Sphingomonas*, *Ramlibacter*, *Herbaspirillum*, *Polaromonas*, and *Azospirillum* were significantly higher, whereas those of *Dokdonella*, *Gemmatirosa*, *Castellaniella*, *Gemmatimonas*, *Chitinophaga*, *Myxococcus*, *Mizugakiibacter*, *Trichoderma*, and *Moritella* were significantly lower in the SA7 soil than in the SF7 sample (Figure 4C). Collectively, these results suggest that although the structure of the main soil microbial community did not change, the abundance of specific species was significantly altered at different time points after pathogen injection.

### Functional annotations and gene taxonomy predictions for the different rhizosphere microbiomes

**Figure 5:**
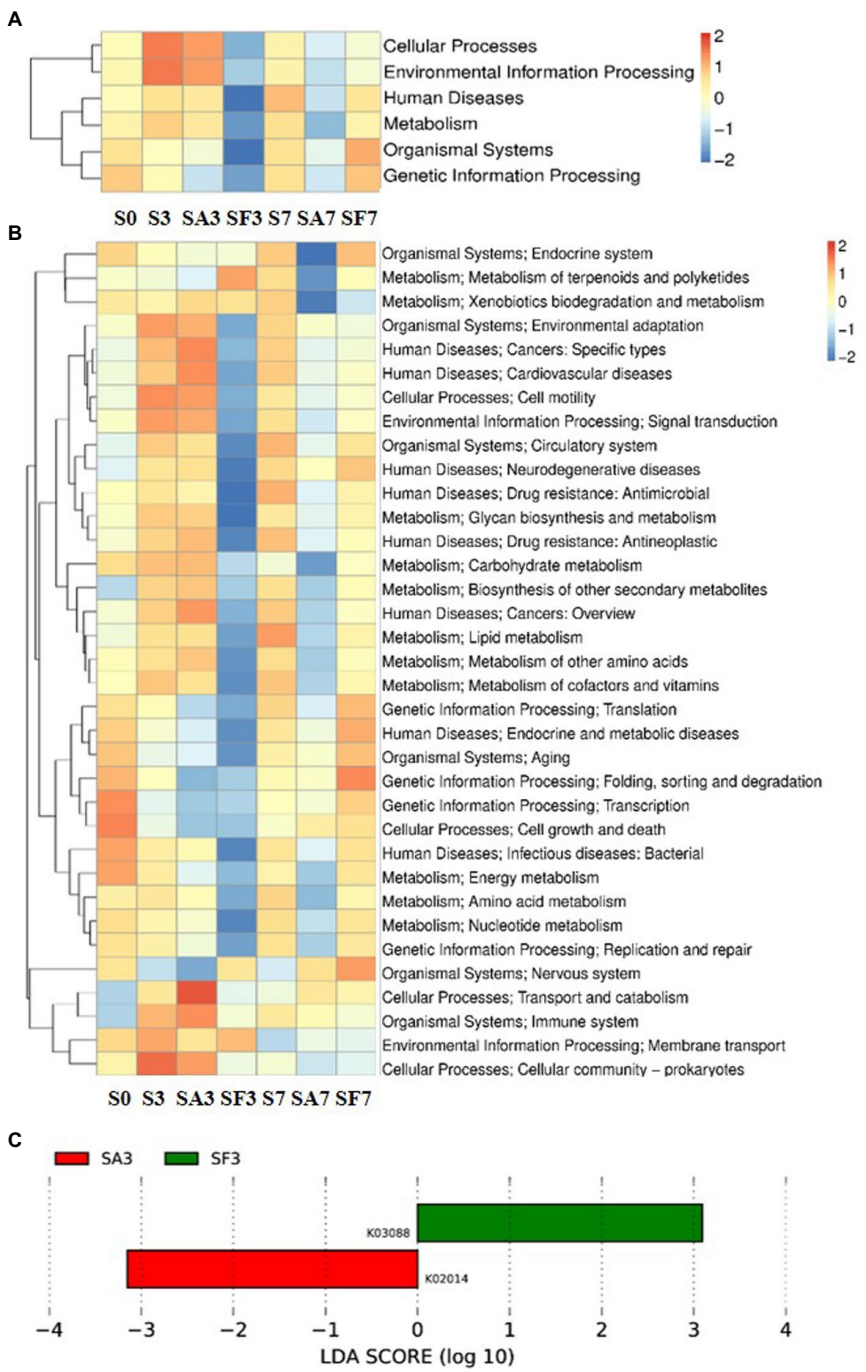
Functional diversity of the microbiomes among different soil samples based on KEGG pathways analysis **(A)** Heatmap of enrichment differences in six major metabolic pathways on level 1 among the samples. **(B)** Heatmap of enrichment differences in metabolic pathways on level 2 among the samples. The *X*-axis represents the sample group names; the *Y*-axis represents the KEGG metabolic pathway annotation information. **(C)** Distribution of linear discriminant analysis (LDA) scores between the SA3 and SF3 KEGG ortholog groups. S0, Mock-inoculation control, before treatment; S3, mock-inoculation control, 3 dpi; S7, mock-inoculation control, 7 dpi; SA3, SA + FON treatment, 3 dpi; SA7, SA + FON treatment, 7 dpi; SF3, FON treatment, 3 dpi; SF7, FON treatment, 7 dpi; FON, *Fusarium oxysporum* f. sp. *niveum*; SA, salicylic acid; dpi, days post-inoculation.

For the SA3 sample, KEGG analysis of the cluster at level 1 revealed the significant activation of four major pathways: environmental information processing, cellular processes, metabolism, and human diseases (Figure 5A). Detailed information on the 43 pathways at cluster level 2 is displayed in Figure 5B. The heatmap of the KEGG ortholog groups (Figure 5C) showed that K06042 (precorrin-8X/cobalt-precorrin-8 methyl mutase), K02055 (putative spermidine/putrescine transport system substrate-binding protein), K16164 (acyl pyruvate hydrolase), and K18930 (D-lactate dehydrogenase) were more highly enriched in the SF3 sample than in the SA3 soil. Furthermore, the distribution of linear discriminant analysis scores between the SA3 and SF3 KEGG ortholog groups indicated that K02014 (iron complex outer membrane receptor protein) was increased in SA3, whereas K03088 (RNA polymerase sigma-70 factor, ECF subfamily) was increased in SF3 (Figure 5C).

**Figure 6:**
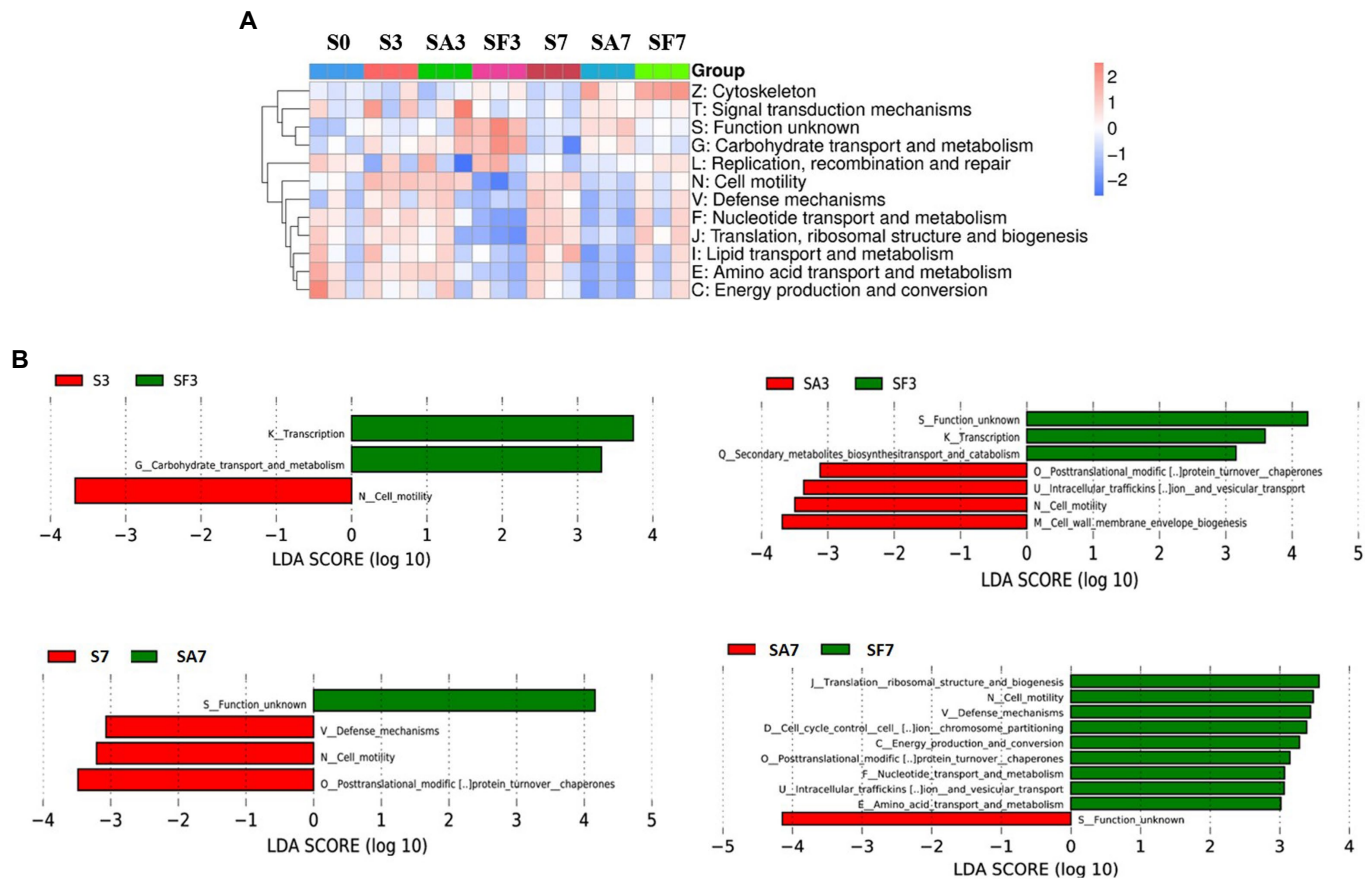
Functional diversity of the microbiomes among different soil samples based on eggNOG metabolic pathway analysis **(A)** Heatmap of enrichment differences in metabolic pathways among the samples. The *X*-axis represents the sample group name; the *Y*-axis represents the eggNOG metabolic pathway annotation information. **(B)** Distribution of LDA scores of functional differences between samples. The *X*-axis represents the LDA score; the *Y*-axis represents the eggNOG metabolic pathway annotation information. Three biological replicates per sample were analyzed. Data are expressed as mean ± SE (*n* = 3). S0, Mock-inoculation control, before treatment; S3, mock-inoculation control, 3 dpi; S7, mock-inoculation control, 7 dpi; SA3, SA + FON treatment, 3 dpi; SA7, SA + FON treatment, 7dpi; SF3, FON treatment, 3 dpi; SF7, FON treatment, 7 dpi. FON, *Fusarium oxysporum* f. sp. *niveum*; SA, salicylic acid; dpi, days post-inoculation.

The heatmap of the eggNOG classification, where the significantly expressed genes were merged into 12 groups, is shown in Figure 6A. Histograms of the distribution of linear discriminant analysis scores for resistance genes with statistically significant differences between the groups are shown in Figure 6B. For instance, there was a significantly higher expression level of genes related to transcription, carbohydrate transport, and metabolism in SF3 than in S3 plants. The genes significantly expressed in SA3 belonged mainly to post-translational; intracellular trafficking, secretion; cell motility; and cell wall: membrane envelope biogenesis. In contrast, those in SF3 belonged primarily to transcription and secondary metabolite biosynthesis. However, SF7 had a significantly higher number of genes related to translation, ribosomal structure, and biogenesis; cell cycle control and cell division; carbohydrate transport and metabolism; intracellular trafficking, secretion; energy production and conversion; defense mechanisms; and replication, recombination, and repair.

**Figure 7:**
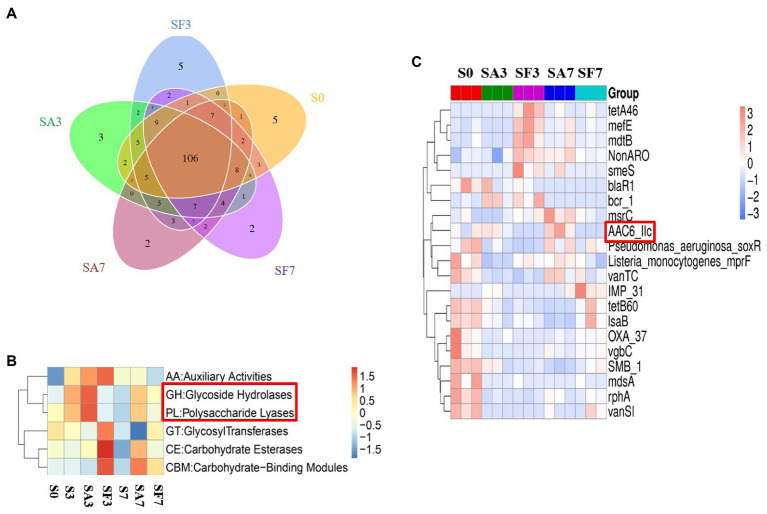
Differential abundance of functional resistance genes among different soil samples **(A)** Venn diagram showing the distribution of functional resistance genes among the five selected sample groups. **(B)** Heatmap of the differences in relative abundance of six CAZy classes among the samples. **(C)** Heatmap of the differences in relative abundance of the antibiotic resistance ontology (ARO) cluster among the samples. S0, Mock-inoculation control, before treatment; S3, mock-inoculation control, 3 dpi; S7, mock-inoculation control, 7 dpi; SA3, SA + FON treatment, 3 dpi; SA7, SA + FON treatment, 7 dpi; SF3, FON treatment, 3 dpi; SF7, FON treatment, 7 dpi. FON, *Fusarium oxysporum* f. sp. *niveum*; SA, salicylic acid; dpi, days post-inoculation.

**Figure 8:**
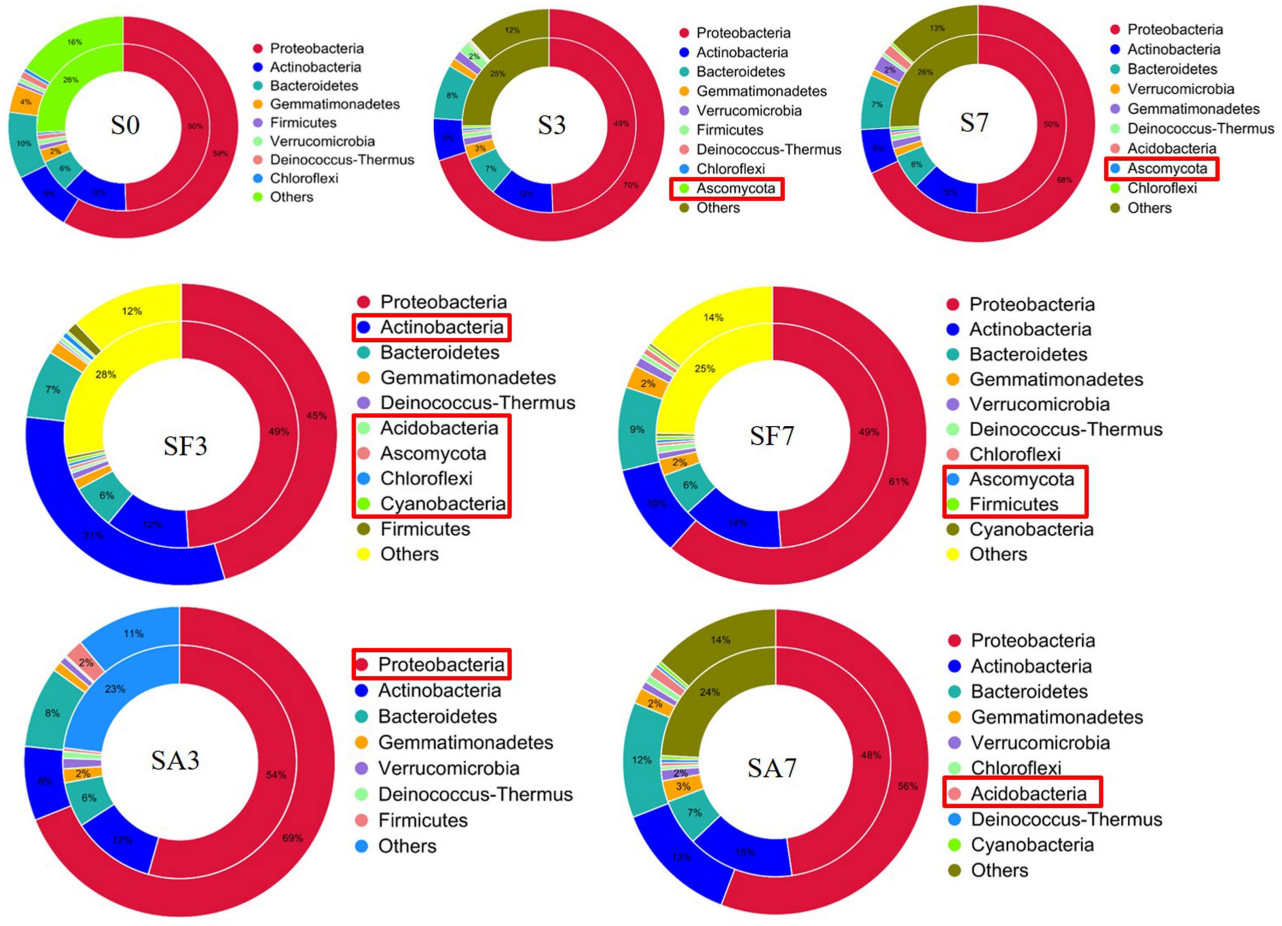
Comparison of the relationship between resistance genes and main phyla in the different soil samples S0, Mock-inoculation control, before treatment; S3, mock-inoculation control, 3 dpi; S7, mock-inoculation control, 7 dpi; SA3, SA + FON treatment, 3 dpi; SA7, SA + FON treatment, 7 dpi; SF3: FON treatment, 3 dpi; SF7, FON treatment, 7 dpi. The inner circle is the phylum distribution for the antibiotic resistance ontology (ARO) cluster, and the outer circle is the phylum distribution for all sample genes in the group. FON, *Fusarium oxysporum* f. sp. *niveum*; SA, salicylic acid; dpi, days post inoculation.

A Venn diagram of the distribution of resistance genes among the five selected samples is shown in Figure 7A. Heatmap analysis of the six CAZy classes indicated that the highly enriched genes in SA3 were in the classes GH: glycoside hydrolases and PL: polysaccharide lyases (Figure 7B). Analysis of the relative abundance of Unigenes showed that the major facilitator superfamily antibiotic efflux pump (*bcr_1*), drug class of aminoglycoside antibiotics, resistance mechanism of antibiotic inactivation (*AAC6_IIC*), and *Serratia* metallo-beta-lactamase (*SMB_1*) genes were highly expressed in SA3, whereas the ATP-binding cassette antibiotic efflux pump (*msrC*), drug class of aminoglycoside antibiotic, resistance mechanism of antibiotic inactivation (*AAC6_IIC*), ATP-binding cassette antibiotic efflux pump, major facilitator superfamily antibiotic efflux pump, resistance-nodulation-cell division antibiotic efflux pump (*Pseudomonas_aeruginosa_soxR*), and defensin-resistant *mprF* (*Listeria_monocytogenes_mprF*) genes were increased in SA7 (Figure 7C). Finally, two-circle graphs (Figure 8) of the main phyla associated with the resistance genes expressed in the soil samples indicated that Proteobacteria were the predominant source in SA3, whereas Chloroflexi, Acidobacteria, and Cyanobacteria expressed most of the resistance genes in SA7. Interestingly, some resistance genes from Ascomycota were observed only in the SF groups and those from Firmicutes only in the SF7 group.

## Discussion

### Exogenous salicylic acid application leads to the enrichment of beneficial microbes in the watermelon rhizosphere

The rhizosphere plays a fundamental role in microbe–microbe and plant–soil–microbe interactions. Microbes and their interactions can extend the capacity of plants for disease resistance and improve their nutrient use efficiency (Berendsen et al., 2018). Emerging evidence indicates that some microbial symbionts communicate with the plant immune system through multiple feedback mechanisms, giving the plant the ability to resist pathogens and to maintain growth and development (Liu et al., 2021). Many studies have emphasized the role of soil microbial communities in enhancing plant growth and health (Aleklett et al., 2018; Toju et al., 2018). For example, Xin et al. found that the plant immune system is required to maintain the normal growth of commensal bacteria in *Arabidopsis* (Xin et al., 2016). Other studies have reported that assemblages of host-specific microbiomes in the rhizosphere are vital for disease resistance. For instance, some disease-resistant crop varieties are enriched in specific sets of bacterial species in the rhizosphere, which contribute to the suppression of pathogens (Delgado-Baquerizo et al., 2018). Another study showed that SA causes changes in the microbiome through allelopathy in wheat (Kong et al., 2020). It is not known how plant roots normally select and maintain a healthy rhizosphere microbiota. SA plays an important role in regulating plant immunity, which is necessary for systemically acquired resistance (Trivedi et al., 2020). In agreement with this theory, our results indicated that although the structure of the main soil microbial community did not change, specific microbes were significantly altered at different time points after pathogen injection. Likewise, we observed that Proteobacteria, the second largest phylum of hydrogenogenic CO oxidizers (Badger and Bek, 2008; Wang and Sugiyama, 2020), accumulated significantly in SA3. Lebeis et al. found that SA could modulate the root microbiome of *A. thaliana*. Specifically, plants with altered SA signaling had root microbiomes that differed from each other in their relative abundance of Proteobacteria as one of the core microbiomes when compared with those of wild-type plants (Lebeis et al., 2015). Furthermore, at the genus level, SA3 had a significantly higher abundance of *Rhodanobacter* than SF3 and SA7 had a higher abundance of *Azospirillum*, *Herbaspirillum*, *Stenotrophomonas*, and *Sphingomonas* than SF7. *Rhodanobacter* is capable of oxidizing ammonia and of denitrification (De Cercq et al., 2006; Huo et al., 2018). Some members of the genus *Azospirillum* exhibit biocontrol activity against phytopathogens and have been used as biofertilizers because of their plant growth-promoting activities, such as biological nitrogen fixation, hormone production, phosphate solubilization, and siderophore production (Mendez-Gomez et al., 2020). *Sphingomonas* is considered an abundant microbial resource for the biodegradation of aromatic compounds, thus showing great potential for environmental protection and industrial production applications because of its high metabolic capacity and multifunctional physiological characteristics (Vorholt et al., 2017). *Stenotrophomonas maltophilia*, which exists widely in water, soil, and animals, is a multidrug-resistant opportunistic pathogen that causes life-threatening infections in immunocompromised individuals (Messiha et al., 2007; Mendes et al., 2013; Hassan and Bano, 2016). *Herbaspirillum* species have been described as closely associated with plants, both endophytically and epiphytically, because their nitrogenase activity promotes plant growth (Wang et al., 2014). For instance, using histochemical analysis, *Herbaspirillum seropedicae* has been shown to colonize the root surfaces and inner tissues of maize, sorghum, wheat, and rice seedlings grown in vermiculite (Ramos et al., 2020).

Therefore, these significantly altered microbial communities confirmed our hypothesis that SA can recruit beneficial microorganisms (such as *Rhodanobacter*, *Sphingomonas*, *and Micromonospora*) in the watermelon rhizosphere. The next question that needs to be answered is whether SA-influenced changes in rhizosphere microflora are beneficial to the antagonism of plants against pathogens, that is, is the increased resistance of plants to pathogens induced directly by SA alone, or by the rhizosphere microbiota, or by both?

### Significantly expressed microbiome genes may interact with salicylic acid signaling to regulate watermelon resistance to disease

Many researchers have found that SA and plant-associated beneficial microorganisms are key candidates for systemic acquired resistance (SAR) and induced systemic resistance (ISR), respectively (Berendsen et al., 2018; Teixeira et al., 2019). Therefore, to elucidate the molecular mechanism through which the rhizosphere microbial community cooperates with the watermelon plant to induce resistance against *Fusarium* wilt, we blasted unique genes to annotate their functions. Surprisingly, genes enriched in environmental information processing, cellular processes, and metabolism pathways were significantly more highly expressed in SA3 than in SF3. Furthermore, K02014 (iron complex outer membrane receptor protein) was expressed at significantly higher levels in SA3 than in SF3. Simultaneously, the genes enriched in the process groups of cell motility and cell wall: membrane envelope biogenesis were expressed more significantly in SA3 than in SF3. These results confirm our hypothesis that the rhizosphere microbiome assemblage is affected by SA signal transduction pathways (Mendes et al., 2011; Chaparro et al., 2014; Vorholt et al., 2017; Delgado-Baquerizo et al., 2018). Notably, the resistance genes from GHs and PLs were highly accumulated in SA3 compared with those in the other soil groups. In particular, *AAC6_IIC* was consistently more significantly accumulated in the SA groups, indicating that it may play an important role in interplay with plant lipid membranes. Furthermore, we noticed that the relative abundance of Proteobacteria corresponding to resistance genes was the highest post-inoculation, which means that more of such genes may come from species in this phylum. Our results confirm the conclusions of other studies that plant genetics and agricultural practices can potentially impose selective pressures on specific microbes and microbial communities. Thus, our findings not only show that SA treatment is beneficial to the antagonism of plants against pathogens but also suggest that, during pathogen infection, the rhizosphere microbiome may play a key role in activating a series of defensive feedback mechanisms in the plant through SA signal transduction pathways.

## Conclusion

In conclusion, our results indicate that exogenous SA treatment can specifically increase some beneficial rhizosphere species that can confer resistance against FON inoculation. The glycoside hydrolase and polysaccharide lyase genes in the microbiome, specifically from a group of beneficial microbes (such as *Rhodanobacter*, *Sphingomonas*, *and Micromonospora*), may potentially induce activation of the plant immune system against *Fusarium* wilt disease and promote plant growth. Our results provide a novel strategy for controlling *Fusarium* wilt in watermelon by manipulating the rhizosphere microbiome through phytohormones, such as exogenous SA treatment. Furthermore, we aim to elucidate the mechanisms underlying the interplay between lipid membrane signaling and SA signal transduction pathways in future studies.

## Data availability statement

The datasets presented in this study can be found in online repositories. The names of the repository/repositories and accession number(s) can be found in the article/Supplementary material.

## Author contributions

FZ and RW: conceptualization. YF, ZW, and FZ: methodology. PW, KY, and FZ: investigation. FZ: writing—original draft preparation. FZ and RW: writing—review and editing and funding acquisition. LX and RW: supervision. All authors contributed to the article and approved the submitted version.

## Funding

This study was financially supported by the National Natural Science Foundation of China (nos. 31671777 and 31871714) to RW, the Natural Science Foundation of Hunan Province, China (no. 2022JJ40213), and the Natural Science Foundation of Changsha City, China (no. kq2202335) to FZ.

## Conflict of interest

The authors declare that the research was conducted in the absence of any commercial or financial relationships that could be construed as a potential conflict of interest.

## Publisher’s note

All claims expressed in this article are solely those of the authors and do not necessarily represent those of their affiliated organizations, or those of the publisher, the editors and the reviewers. Any product that may be evaluated in this article, or claim that may be made by its manufacturer, is not guaranteed or endorsed by the publisher.

## Abbreviations

ARO: antibiotic resistance ontology
dpi: days post-inoculation
FON: *Fusarium oxysporum* f. sp. *niveum*
hpi: hours post-inoculation
MDA: malondialdehyde
PAL: phenylalanine ammonia lyase
POD: peroxidase
SA: salicylic acid

## References

[ref1] Aleklett K. Kiers E. T. Ohlsson P. Shimizu T. S. Caldas V. E. Hammer E. C. (2018). Build your own soil: exploring microfluidics to create microbial habitat structures. ISME J. 12, 312–319. doi: 10.1038/ismej.2017.184, PMID: 29135971 PMC5776464

[ref2] Badger M. R. Bek E. J. (2008). Multiple rubisco forms in proteobacteria: their functional significance in relation to co2 acquisition by the cbb cycle. J. Exp. Bot. 59, 1525–1541. doi: 10.1093/jxb/erm297 18245799

[ref3] Berendsen R. L. Vismans G. Yu K. Song Y. de Jonge R. Burgman W. P.. (2018). Disease-induced assemblage of a plant-beneficial bacterial consortium. ISME J. 12, 1496–1507. doi: 10.1038/s41396-018-0093-1, PMID: 29520025 PMC5956071

[ref4] Chaparro J. M. Badri D. V. Vivanco J. M. (2014). Rhizosphere microbiome assemblage is affected by plant development. ISME J. 8, 790–803. doi: 10.1038/ismej.2013.196, PMID: 24196324 PMC3960538

[ref5] Chen T. Nomura K. Wang X. Sohrabi R. Xu J. Yao L.. (2020). A plant genetic network for preventing dysbiosis in the phyllosphere. Nature 580, 653–657. doi: 10.1038/s41586-020-2185-0, PMID: 32350464 PMC7197412

[ref6] De Cercq D. Van Tappen S. Cleenwerck I. Ceustermans A. Swings J. Coosemans J.. (2006). Rhodanobacter spathiphylli sp. nov., a gammaproteobacterium isolated from the roots of Spathiphyllum plants grown in a compost-amended potting mix. Int. J. Syst. Evol. Microbiol. 56, 1755–1759. doi: 10.1099/ijs.0.64131-0 16902003

[ref7] Delgado-Baquerizo M. Oliverio A. M. Brewer T. E. Benavent-Gonzalez A. Eldridge D. J. Bardgett R. D.. (2018). A global atlas of the dominant bacteria found in soil. Science 359, 320–325. doi: 10.1126/science.aap9516, PMID: 29348236

[ref8] Everts K. L. Himmelstein J. C. (2015). Fusarium wilt of watermelon: towards sustainable management of a re-emerging plant disease. Crop Prot. 73, 93–99. doi: 10.1016/j.cropro.2015.02.019

[ref9] Hassan T. U. Bano A. (2016). Comparitive effects of wild type Stenotrophomonas maltophilia and its IAA deficient mutants on wheat. Plant Biol. 18, 835–841. doi: 10.1111/plb.12477, PMID: 27263526

[ref10] Heintz C. Mair W. (2014). You are what you host: microbiome modulation of the aging process. Cells 156, 408–411. doi: 10.1016/j.cell.2014.01.025, PMID: 24485451 PMC3956044

[ref11] Huo Y. Kang J. P. Park J. K. Li J. Chen L. Yang D. C. (2018). Rhodanobacter ginsengiterrae sp. nov., an antagonistic bacterium against root rot fungal pathogen fusarium solani, isolated from ginseng rhizospheric soil. Arch. Microbiol. 200, 1457–1463. doi: 10.1007/s00203-018-1560-9, PMID: 30116848

[ref12] Kong H. G. Song G. C. Sim H. J. Ryu C. M. (2020). Achieving similar root microbiota composition in neighbouring plants through airborne signalling. ISME J. 15, 397–408. doi: 10.1038/s41396-020-00759-z, PMID: 32973341 PMC8027813

[ref13] Lebeis S. L. Paredes S. H. Lundberg D. S. Breakfield N. Gehring J. McDonald M.. (2015). Salicylic acid modulates colonization of the root microbiome by specific bacterial taxa. Science 349, 860–864. doi: 10.1126/SCIENCE.AAA8764, PMID: 26184915

[ref14] Levy A. Salas Gonzalez I. Mittelviefhaus M. Clingenpeel S. Herrera Paredes S. Miao J.. (2017). Genomic features of bacterial adaptation to plants. Nat. Genet. 50, 138–150. doi: 10.1038/s41588-017-0012-9, PMID: 29255260 PMC5957079

[ref15] Li C. Fu X. Zhou X. Liu S. Xia Y. Li N.. (2019). Treatment with wheat root exudates and soil microorganisms from wheat/watermelon companion cropping can induce watermelon disease resistance against *fusarium oxysporum f. sp. niveum*. Plant Dis. 103, 1693–1702. doi: 10.1094/PDIS-08-18-1387-RE, PMID: 31106703

[ref16] Liu H. Li J. Carvalhais L. C. Percy C. D. Prakash Verma J. Schenk P. M.. (2021). Evidence for the plant recruitment of beneficial microbes to suppress soil-borne pathogens. New Phytol. 229, 2873–2885. doi: 10.1111/nph.17057, PMID: 33131088

[ref17] Lü G. Guo S. Zhang H. Geng L. Song F. Fei Z.. (2011). Transcriptional profiling of watermelon during its incompatible interaction with fusarium oxysporum f. sp. niveum. Eur. J. Plant Pathol. 131, 585–601. doi: 10.1007/s10658-011-9833-z

[ref18] Lv H. Cao H. Nawaz M. A. Sohail H. Huang Y. Cheng F.. (2018). Wheat intercropping enhances the resistance of watermelon to fusarium wilt. Front. Plant Sci. 9:696. doi: 10.3389/fpls.2018.00696, PMID: 29887873 PMC5980984

[ref19] Mendes R. Garbeva P. Raaijmakers J. M. (2013). The rhizosphere microbiome: significance of plant beneficial, plant pathogenic, and human pathogenic microorganisms. FEMS Microbiol. Rev. 37, 634–663. doi: 10.1111/1574-6976.12028, PMID: 23790204

[ref20] Mendes R. Kruijt M. de Bruijn I. Dekkers E. van der Voort M. Schneider J. H.. (2011). Deciphering the rhizosphere microbiome for disease-suppressive bacteria. Science 332, 1097–1100. doi: 10.1126/science.1203980, PMID: 21551032

[ref21] Mendez-Gomez M. Castro-Mercado E. Pena-Uribe C. A. L Cruz H. R. Lopez-Bucio J. Garcia-Pineda E. (2020). Azospirillum brasilense Sp245 lipopolysaccharides induce target of rapamycin signaling and growth in Arabidopsis thaliana. J. Plant Physiol. 253:153270. doi: 10.1016/j.jplph.2020.153270, PMID: 32919283

[ref22] Messiha N. A. S. Diepeningen A. D. V. Farag N. S. Abdallah S. A. Janse J. D. Bruggen A. H. C. V. (2007). Stenotrophomonas maltophilia: A new potential biocontrol agent *of Ralstonia solanacearum*, causal agent of potato brown rot. Eur. J. Plant Pathol. 118, 211–225. doi: 10.1007/s10658-007-9136-6

[ref23] Ramos A. C. Melo J. de Souza S. B. Bertolazi A. A. Silva R. A. Rodrigues W. P.. (2020). Inoculation with the endophytic bacterium Herbaspirillum seropedicae promotes growth, nutrient uptake and photosynthetic efficiency in rice. Planta 252:87. doi: 10.1007/s00425-020-03496-x, PMID: 33057912

[ref24] Ren L. Huo H. Zhang F. Hao W. Xiao L. Dong C.. (2016). The components of rice and watermelon root exudates and their effects on pathogenic fungus and watermelon defense. Plant Signal. Behav. 11:e1187357. doi: 10.1080/15592324.2016.1187357, PMID: 27217091 PMC4977455

[ref25] Teixeira P. J. P. Colaianni N. R. Fitzpatrick C. R. Dangl J. L. (2019). Beyond pathogens: microbiota interactions with the plant immune system. Curr. Opin. Microbiol. 49, 7–17. doi: 10.1016/j.mib.2019.08.003, PMID: 31563068

[ref26] Toju H. Peay K. G. Yamamichi M. Narisawa K. Hiruma K. Naito K.. (2018). Core microbiomes for sustainable agroecosystems. Nat. Plants 4, 247–257. doi: 10.1038/s41477-018-0139-4, PMID: 29725101

[ref27] Trivedi P. Leach J. E. Tringe S. G. Sa T. Singh B. K. (2020). Plant-microbiome interactions: from community assembly to plant health. Nat. Rev. Microbiol. 18, 607–621. doi: 10.1038/s41579-020-0412-1, PMID: 32788714

[ref28] Vorholt J. A. Vogel C. Carlström C. I. Müller D. B. (2017). Establishing causality: opportunities of synthetic communities for plant microbiome research. Cell Host Microbe 22, 142–155. doi: 10.1016/j.chom.2017.07.004, PMID: 28799900

[ref29] Wang X. Cao Y. Tang X. Ma X. Gao J. Zhang X. (2014). Rice endogenous nitrogen fixing and growth promoting bacterium Herbaspirillum seropedicae DX35. Acta Microbiol Sin. 54, 292–298. doi: 10.13343/j.cnki.wsxb.2014.03.006, PMID: 24984521

[ref30] Wang B. Sugiyama S. (2020). Phylogenetic signal of host plants in the bacterial and fungal root microbiomes of cultivated angiosperms. Plant J. 104, 522–531. doi: 10.1111/tpj.14943, PMID: 32744366

[ref31] Xin X. F. Nomura K. Aung K. Velasquez A. C. Yao J. Boutrot F.. (2016). Bacteria establish an aqueous living space in plants crucial for virulence. Nature 539, 524–529. doi: 10.1038/nature20166, PMID: 27882964 PMC5135018

[ref32] Xu W. Wang Z. Wu F. (2015). Companion cropping with wheat increases resistance to fusarium wilt in watermelon and the roles of root exudates in watermelon root growth. Physiol. Mol. Plant Pathol. 90, 12–20. doi: 10.1016/j.pmpp.2015.02.003

[ref33] Yang L. Li B. Zheng X. Y. Li J. Yang M. Dong X.. (2015). Salicylic acid biosynthesis is enhanced and contributes to increased biotrophic pathogen resistance in Arabidopsis hybrids. Nat. Commun. 6:7309. doi: 10.1038/ncomms8309, PMID: 26065719 PMC4490401

[ref34] Zhang Y. Li X. (2019). Salicylic acid: biosynthesis, perception, and contributions to plant immunity. Curr. Opin. Plant Biol. 50, 29–36. doi: 10.1016/j.pbi.2019.02.004, PMID: 30901692

[ref35] Zhu F. Tian C. Zhang Y. Xiao J. Wei L. Liang Z. (2018). Effects of different fertilization treatments on soil microbial community structure and the occurrence of watermelon wilt. Chin. J. Biol. Control 34, 589–597. doi: 10.16409/j.cnki.2095-039x.2018.04.014

[ref36] Zhu F. Wang Z. Fang Y. Tong J. Xiang J. Yang K.. (2022a). Study on the role of Phytohormones in resistance to watermelon fusarium wilt. Plants 11:156. doi: 10.3390/plants11020156, PMID: 35050045 PMC8781552

[ref37] Zhu F. Wang Z. Su W. Tong J. Fang Y. Luo Z.. (2022b). Study on the role of salicylic acid in watermelon-resistant fusarium wilt under different growth conditions. Plants 11:293. doi: 10.3390/plants11030293, PMID: 35161274 PMC8839013

[ref38] Zhu F. Xiao J. Zhang Y. Wei L. Liang Z. (2020). Dazomet application suppressed watermelon wilt by the altered soil microbial community. Sci. Rep. 10:21668. doi: 10.1038/s41598-020-78839-5, PMID: 33303943 PMC7730150

[ref39] Zhu F. Zhang Y. Xiao J. Wei L. Liang Z. (2019). Regulation of soil microbial community structures and watermelon fusarium wilt by using bio-organic fertilizer. Acta Microbiol Sin. 59, 2323–2333. doi: 10.13343/j.cnki.wsxb.20190038

