# Supplementary material for: Salicylic acid remodeling of the rhizosphere microbiome induces watermelon root resistance against Fusarium oxysporum f. sp. niveum infection
Source: Front Microbiol. 2022 Sep 23;13:1015038. doi: 10.3389/fmicb.2022.1015038 (PMC9539938; doi:10.3389/fmicb.2022.1015038)

Supplementary Material

Salicylic Acid Remodeling of the Rhizosphere Microbiome Induces Watermelon Root Resistance Against *Fusarium oxysporum* f. sp. *niveum* Infection

Supplementary Figure 1. The Sequencing and metagenome assembly. A, Gene length distribution; B, Box plot of gene number difference between groups; C, Venn Graph. Note: S0 (Mock-inoculation control, before treatment), S3 (Mock-inoculation control, 3 dpi), S7 (Mock-inoculation control, 7dpi), SA3 (SA+FON treatment, 3dpi), SA7 (SA+FON treatment, 7dpi), SF3 (FON treatment, 3dpi), SF7 (FON treatment, 7dpi). Three biological replicates per samples were analyzed. Data are expressed as mean ± SE (n = 3).

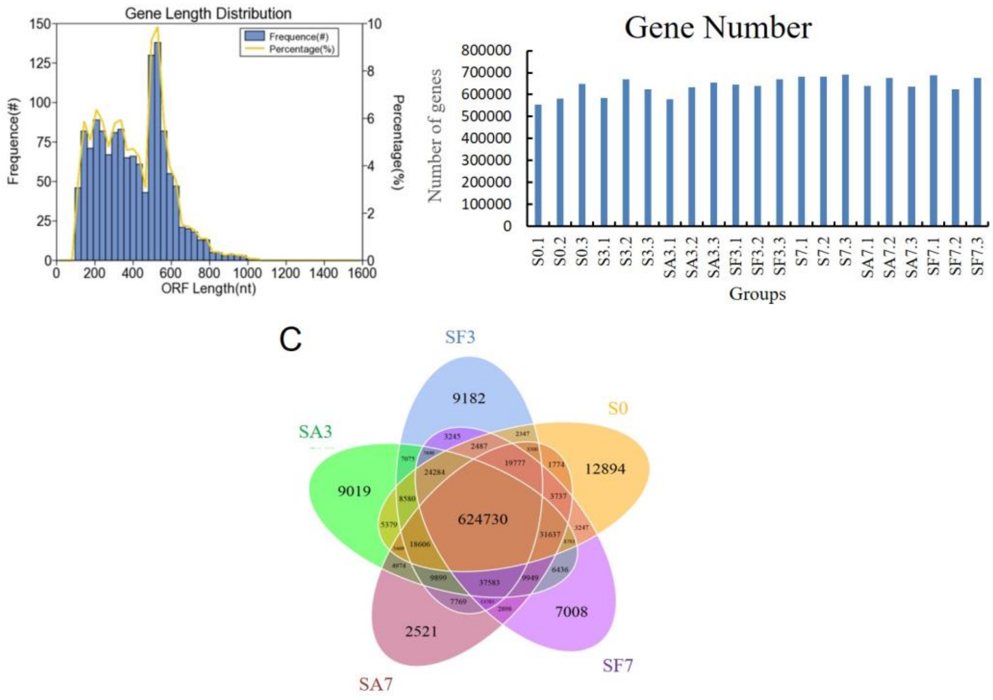

Supplement: Supplementary file 1 [file Data_Sheet_1.PDF]
